# Utilization Trends of Dual GIP/GLP-1 Receptor Agonist, Newer Glucose-Lowering Medications, and Anti-Obesity Medications Among Patients With Chronic Kidney Disease With and Without Type 2 Diabetes

**DOI:** 10.1016/j.xkme.2025.101013

**Published:** 2025-04-19

**Authors:** Panupong Hansrivijit, Janinne Ortega-Montiel, Deborah J. Wexler, Elisabetta Patorno, Julie M. Paik

**Affiliations:** 1 Division of Pharmacoepidemiology & Pharmacoeconomics, Brigham and Women’s Hospital, Boston, MA; 2 Division of Renal (Kidney) Medicine, Brigham and Women’s Hospital, Boston, MA; 3 Diabetes Center, Massachusetts General Hospital, Boston, MA; 4 Harvard Medical School, Boston, MA; 5 New England Geriatric Research Education and Clinical Center, VA Boston Healthcare System, Boston, MA

**Keywords:** GIP/GLP-1, chronic kidney disease, type 2 diabetes, adult obesity, prescribing trends, SGLT2 inhibitors, GLP-1 receptor agonists

## Abstract

**Rationale & Objective:**

Tirzepatide, a dual GIP/GLP-1 receptor agonist, has been approved for type 2 diabetes (T2D) and obesity. However, the real-world utilization of tirzepatide remains unexplored, particularly in patients with chronic kidney disease (CKD), where the prevalence of T2D and obesity is high. This study aimed to describe the utilization trends of tirzepatide, glucose-lowering medications (GLMs), and anti-obesity medications (AOMs) in patients with CKD, with and without T2D.

**Study Design:**

A population-based, observational cohort study.

**Setting & Participants:**

Patients with CKD, with and without T2D, were identified from a large US health insurance claims database (from January 1, 2022 to September 30, 2023).

**Exposures:**

Tirzepatide, other GLMs, and AOMs.

**Outcomes:**

Medication utilization trends and patient characteristics. Any users were defined as those with prescription claims, and incident users as those with no previous dispensing within 365 days.

**Analytical Approach:**

Longitudinal trends were assessed by 1-month intervals from January 1, 2022 to September 30, 2023.

**Results:**

Among 455,047 patients with CKD and T2D, tirzepatide any users increased to 4.8% in September 2023, while incident users rose from 0.8% to 8.6%. Sodium glucose cotransporter-2 inhibitors remained the most initiated GLM. Tirzepatide initiators had higher rates of obesity (32.5%), and morbid obesity (44.1%) when compared with other GLMs. Among 5,978 patients with CKD without diabetes, weekly semaglutide ≤2 mg was the most initiated AOM, followed by tirzepatide. Incident users of tirzepatide rose from 0.6% in June 2022 to 23.5% in September 2023. Clinical characteristics were similar between semaglutide ≤2 mg versus tirzepatide initiators.

**Limitations:**

The study period ended before tirzepatide’s approval for weight management (November 2023).

**Conclusions:**

Our study indicates rapidly shifting trends in tirzepatide uptake among patients with CKD both with and without diabetes. The uptake of tirzepatide is expected to increase further. Future studies on the comparative effectiveness and safety of tirzepatide in patients with CKD are warranted.

## Plain Language Summary

Tirzepatide, a dual GIP/GLP-1 receptor agonist, has been approved for glycemic control and weight management, but its utilization in the real-world settings among patients with chronic kidney disease (CKD) is unknown, where the prevalence of type 2 diabetes and obesity is high. In this study, we examined the utilization trends of tirzepatide, other glucose-lowering medications, and anti-obesity medications (AOMs) from January 1, 2022 to September 30, 2023 using a large US health insurance database. We found that the use of tirzepatide has been rapidly rising as a GLM and AOM among patients with CKD and is expected to increase further. Future studies on the effectiveness and safety of tirzepatide against other glucose-lowering medications or AOMs in patients with CKD are needed.

Tirzepatide, a dual agonist of the glucose-dependent insulinotropic polypeptide (GIP) and glucagon-like peptide-1 (GLP-1) receptors, was approved by the United States Food and Drug Administration (US FDA) on May 13, 2022 for glycemic control in adults with type 2 diabetes (T2D).^1^ Although tirzepatide was not approved by the US FDA for weight management until November 2023,^2^ the effect of tirzepatide on weight reduction was evident since the SURPASS program.^3^, ^4^, ^5^ The effects of tirzepatide on weight management were then further demonstrated in the SURMOUNT series trials,^6^^,^^7^ which led to the FDA approval of tirzepatide for weight loss in November 2023 for adults with obesity, overweight, and obesity-related complications.^2^^,^^8^

However, the use of tirzepatide in patients with chronic kidney disease (CKD) remains understudied due to limited number of patients with CKD in the clinicals trials. Although the SURPASS trial series included patients with estimated glomerular filtration rate (eGFR) >30 mL/min/1.73m^2^, only a small proportion of patients had pre-existing CKD as defined by eGFR <60 mL/min/1.73m.^3^, ^4^, ^5^ For example, in the SURPASS-2 trial,^4^ only 10% of patients assigned to tirzepatide and 4% of patients assigned to semaglutide had eGFR < 60 mL/min/1.73m^2^.

Moreover, T2D and obesity, the 2 main FDA-approved indications for tirzepatide, pose a substantial burden on patients with CKD. Patients with CKD have a higher prevalence of T2D, and obesity.^9^ Among patients with CKD, the prevalence of T2D was 35.2% compared with 13.3% for patients without CKD. Similarly, obesity was more prevalent among patients with CKD (49.1%) compared with patients without CKD (41.7%) in the United States.^9^ Given the substantial burden of T2D and obesity in patients with CKD, interventions that address T2D or obesity, such as tirzepatide, some glucose-lowering medications (GLMs) and anti-obesity medications (AOMs), are expected to positively impact CKD outcomes. A key step in designing the comparative effectiveness of tirzepatide in patients with CKD is to study the utilization trends of tirzepatide, GLMs, and AOMs among the population with CKD.

We conducted this cohort study to assess the uptake of tirzepatide among patients with CKD after FDA approval for glycemic control, in comparison with other GLMs and AOMs. In addition, we analyzed patients’ clinical characteristics, distinguishing incident users of tirzepatide from those of other GLMs and AOMs.

## Methods

### Data Sources

We used data from a large US commercial health insurance claims database, Optum’s de-identified Clinformatics Data Mart Database (Clinformatics) from January 1, 2022 to September 30, 2023. Clinformatics comprises commercial health plan members and Medicare advantage members across all 50 states and the District of Columbia. Longitudinal information on clinical characteristics, procedures, inpatient and outpatient diagnoses, and outpatient prescription dispensing are available for all enrollees. Laboratory results are available in about 40% of enrollees. This study was approved by the Mass General Brigham institutional review board, and a data use agreement was in place.

### Study Design

This is a descriptive cohort study delineating the utilization trends of tirzepatide, newer GLMs, such as sodium glucose cotransporter-2 inhibitors (SGLT2i) and glucagon-like peptide-1 receptor agonists (GLP-1RA), and AOMs among patients with CKD with and without T2D from January 1, 2022 to September 30, 2023. We selected January 1, 2022, as the first date of cohort entry because tirzepatide was approved by the US FDA in year 2022 (May 2022).

### Study Population

We divided the study into 2 cohorts. The first cohort, CKD and T2D, included patients with International Classification of Diseases diagnosis codes for CKD^10^ and T2D^11^ who were prescribed at least one GLM from January 1, 2022 to September 30, 2023. The second cohort, CKD without diabetes, included patients with International Classification of Diseases diagnosis codes for CKD^10^ who were prescribed at least one AOM from January 1, 2022 to September 30, 2023. In both cohorts, we excluded patients age <18 years, patients with less than 365 days of continuous health plan enrollment before cohort entry date patients with type 1 diabetes, secondary or gestational diabetes diagnoses. In the cohort of patients with CKD without diabetes, we also excluded patients taking a GLM (insulin, sulfonylureas [SU], dipeptidyl peptidase-4 inhibitors [DPP4i], thiazolidinediones [TZD], α-glucosidase inhibitors, meglitinide, and pramlintide) as the use of these medications could suggest an underlying diagnosis of diabetes. We allowed patients taking metformin, SGLT2i, and GLP-1RA into the cohort, as these medications can be prescribed to patients who do not have diabetes. The cohort entry date was defined by the date of the first prescription of any GLMs (in the CKD and T2D cohort) or AOMs (in the CKD without diabetes cohort) from January 1, 2022, to September 30, 2023.

### CKD and T2D Cohort: Any Users and Incident Users of Tirzepatide and Other GLMs

In patients with CKD and T2D, any users were defined as patients who had prescription claims for a given GLM from January 1, 2022, to September 30, 2023, regardless of previous use history. Incident users were defined as new initiators of a given GLM without previous use within 365 days. The assessed GLM classes included tirzepatide, SGLT2i, GLP-1RA, insulins, metformin, second-generation SU, DPP4i, TZD, and miscellaneous agents (first-generation SU, α-glucosidase inhibitors, meglitinide, and pramlintide).

For any users in the cohort of patients with CKD and T2D, percentages of any users of a given GLM were calculated monthly from January 1, 2022, to September 30, 2023, by dividing the number of any users of a given GLM class by the total number of patients using any GLMs during that month. For incident users in the cohort of patients with CKD and T2D, percentages of incident users of a given GLM were calculated monthly from January 1, 2022, to September 30, 2023, by dividing the number of incident users of a given GLM class by the total number of patients initiated on any GLMs during that month.

### CKD Without Diabetes Cohort: Any Users and Incident Users of Tirzepatide and Other AOMs

In patients with CKD without diabetes, any users and incident users of AOMs were assessed in 1-month intervals from January 1, 2022, to September 30, 2023. The studied AOMs included tirzepatide, GLP-1RA, and others (phentermine, naltrexone, bupropion, benzphentamine, diethylpropion, phendimetrazine, and orlistat). We evaluated the use of individual GLP-1RA because some GLP-1RA are used off-label for weight management. These GLP-1RA included subcutaneous (SQ) semaglutide 2.4 mg weekly (Wegovy), SQ emaglutide ≤2 mg weekly (Ozempic), oral semaglutide (Rybelsus), dulaglutide (Trulicity), liraglutide <1.8 mg (Victoza), and SQ liraglutide 3 mg weekly (Saxenda).

For any users in the cohort of patients with CKD without diabetes, percentages of any users of a given AOM were calculated monthly from January 1, 2022, to September 30, 2023, by dividing the number of any users of a given AOM class by the total number of patients using any AOMs during that month. For incident users in the cohort of patients with CKD without diabetes, percentages of incident users of a given AOM were calculated monthly from January 1, 2022, to September 30, 2023 by dividing the number of incident users of a given AOM class by the total number of patients initiated on any given AOMs during that month.

### Clinical Characteristics

We reported the clinical characteristics of incident users of tirzepatide, SGLT2i, and GLP-1RA, as these medications are increasingly prescribed for patients with CKD, from January 1, 2022, to September 30, 2023. The clinical characteristics were assessed during the 365 days before the cohort entry date. These include demographics, comorbid condition score,^12^ frailty score,^13^ diabetic, metabolic, cardiovascular, renal, and gastrointestinal comorbid conditions, medication use, and health care utilization.^14^

### Statistical Analyses

Any users and incident users of each GLM and AOM are reported as percentages. For clinical characteristics, categorical variables are reported as numbers and percentages, with the total sample size as the denominator. Continuous variables are reported as means ± standard deviation (SD). All analyses were performed using Aetion Evidence Platform version 4.92.0 (Aetion, Inc) and R version 2023.06.0+421 (Posit Software, PBC).

## Results

### CKD and T2D Cohort: Utilization Trends of Tirzepatide and Other GLMs

**Figure 1:**
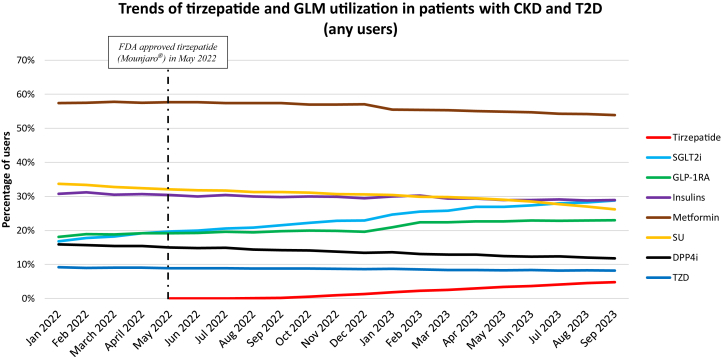
Trends of any use of tirzepatide and glucose-lowering medications (GLMs) among patients with CKD and T2D. Denominator is patients prescribed any GLMs for a given month. CKD, chronic kidney disease; T2D, type 2 diabetes.

We identified 455,047 patients with CKD and T2D who were prescribed at least 1 GLM from January 1, 2022 to September 30, 2023 (Table 1 and Table S1). There was a steady increase in the use of tirzepatide from September 2022 onward, reaching 4.8% of any users of GLM by the end of September 2023 (Fig 1). Any users of SGLT2i and GLP-1RA accounted for nearly 28.8% and 23.0% of total GLM use, respectively, by September 2023. Metformin showed the highest percentage of any users (between 50% and 60%) over the study period. The percentage of insulin users (any users) remained around 30% throughout the study period. Any users of SU and DPP4i gradually declined over the study period (33.7% to 26.2% for SU, 15.9% to 11.8% for DPP4i).

**Table 1:** Clinical Characteristics of Users of Tirzepatide, Newer GLMs, and AOMs Among Patients with CKD and T2D and Without Diabetes From January 1, 2022, to September 30, 2023

Clinical Characteristics	CKD and T2D Cohort			CKD without diabetes Cohort^a^	
	Tirzepatide	SGLT2i	GLP-1RA^b^	Tirzepatide	Semaglutide ≤2 mg
Number of patients	10,661	60,591	39,597	909	2,719
Demographics					
Age (y), mean ± SD	65.9 ± 9.7	72.7 ± 8.8	69.4 ± 9.1	65.4 ± 10.1	66.8 ± 9.3
Gender–female; n (%)	6,165 (57.8%)	27,748 (45.8%)	21,938 (55.4%)	671 (73.8%)	2,025 (74.5%)
Race categories, n (%)					
White	6,845 (64.2%)	34,821 (57.5%)	23,898 (60.4%)	676 (74.4%)	1,946 (71.6%)
Black	1,653 (15.5%)	9,744 (16.1%)	5,878 (14.8%)	98 (10.8%)	350 (12.9%)
Asian	178 (1.7%)	2,546 (4.2%)	1,025 (2.6%)	3 (0.3%)	26 (1.0%)
Hispanic	1,217 (11.4%)	8,508 (14.0%)	5,422 (13.7%)	77 (8.5%)	218 (8.0%)
Others^c^	768 (7.2%)	4,972 (8.2%)	3,374 (8.5%)	55 (6.1%)	179 (6.6%)
Mean combined comorbid condition score (±SD)	3.5 ± 2.8	4.5 ± 3.4	3.7 ± 3.0	2.3 ± 2.5	2.4 ± 2.6
Frailty score, n (%)					
0.00-0.14 (robust)	3,157 (29.6%)	17,146 (28.3%)	12,159 (30.7%)	306 (33.7%)	912 (33.5%)
0.15-0.24 (pre-frail)	6,139 (57.6%)	33,196 (54.8%)	21,825 (55.1%)	521 (57.3%)	1,564 (57.5%)
≥ 0.25 (frail)	1,365 (12.8%)	10,249 (16.9%)	5,613 (14.2%)	82 (9.0%)	243 (8.9%)
Diabetes comorbid conditions					
Diabetic nephropathy; n (%)	6,380 (59.8%)	40,024 (66.1%)	25,092 (63.4%)	-	-
Diabetic neuropathy; n (%)	3,712 (34.8%)	19,399 (32.0%)	14,005 (35.4%)	-	-
Diabetic retinopathy; n (%)	1,484 (13.9%)	9,125 (15.1%)	5,944 (15.0%)	-	-
Diabetic ketoacidosis; n (%)	48 (0.5%)	312 (0.5%)	286 (0.7%)	-	-
Hypoglycemia; n (%)	421 (3.9%)	2,785 (4.6%)	1,826 (4.6%)	-	-
Mean HbA1c; % (± SD)^d^	7.41 ± 1.51	7.41 ± 1.49	7.73 ± 1.59	-	-
Metabolic comorbid conditions^e^					
Underweight or normal weight (BMI < 25 kg/m^2^); n (%)	134 (1.3%)	2,991 (4.9%)	824 (2.1%)	-	19 (0.7%)
Overweight (BMI 25-29.9 kg/m^2^); n (%)	967 (9.1%)	8,578 (14.2%)	4,356 (11.0%)	99 (10.9%)	264 (9.7%)
Obese (BMI 30-39.9 kg/m^2^); n (%)	3,562 (33.4%)	15,357 (25.3%)	11,682 (29.5%)	343 (37.7%)	1,004 (36.9%)
Morbid obese (BMI ≥ 40 kg/m^2^); n (%)	4,784 (44.9%)	12,610 (20.8%)	13,055 (33.0%)	444 (48.8%)	1,359 (50.0%)
Cardiovascular comorbid conditions					
Hypertension; n (%)	9,884 (92.7%)	57,225 (94.4%)	36,575 (92.4%)	749 (82.4%)	2,270 (83.5%)
Hyperlipidemia; n (%)	9,389 (88.1%)	53,303 (88.0%)	34,200 (86.4%)	679 (74.7%)	2,008 (73.9%)
Coronary atherosclerosis; n (%)	2,894 (27.1%)	23,635 (39.0%)	11,403 (28.8%)	178 (19.6%)	554 (20.4%)
Congestive heart failure; n (%)	2,265 (21.2%)	22,074 (36.4%)	8,916 (22.5%)	132 (14.5%)	431 (15.9%)
Atrial fibrillation; n (%)	1,348 (12.6%)	14,202 (23.4%)	5,716 (14.4%)	108 (11.9%)	335 (12.3%)
Ischemic stroke; n (%)	995 (9.3%)	8,721 (14.4%)	4,409 (11.1%)	78 (8.6%)	183 (6.7%)
Peripheral arterial disease; n (%)	1,370 (12.9%)	10,943 (18.1%)	5,915 (14.9%)	92 (10.1%)	262 (9.6%)
Smoking; n (%)	3,131 (29.4%)	19,432 (32.1%)	11,567 (29.2%)	285 (31.4%)	914 (33.6%)
Renal comorbid conditions					
CKD stage 1-2; n (%)	2,284 (21.4%)	11,162 (18.4%)	7,748 (19.6%)	188 (20.7%)	458 (16.8%)
CKD stage 3a-4; n (%)	5,825 (54.6%)	41,483 (68.5%)	23,241 (58.7%)	472 (51.9%)	1,504 (55.3%)
CKD stage 5 and dialysis; n (%)	273 (2.6%)	1,178 (1.9%)	1,209 (3.1%)	-	43 (1.6%)
CKD unspecified; n (%)	2,283 (21.4%)	6,794 (11.2%)	7,367 (18.6%)	238 (26.2%)	716 (26.3%)
Proteinuria; n (%)	1,347 (12.6%)	10,420 (17.2%)	5,120 (12.9%)	52 (5.7%)	154 (5.7%)
Hyperkalemia; n (%)	670 (6.3%)	6,441 (10.6%)	3,269 (8.3%)	27 (3.0%)	87 (3.2%)
Acute kidney injury; n (%)	1,420 (13.3%)	13,824 (22.8%)	6,189 (15.6%)	91 (10.0%)	239 (8.8%)
Mean eGFR (mL/min/1.73m2) (± SD)^f^	58.61 ± 20.63	51.08 ± 18.07	55.49 ± 20.05	59.55 ± 16.54	58.58 ± 16.87
Urinary tract infection; n (%)	1,876 (17.6%)	10,255 (16.9%)	6,908 (17.4%)	155 (17.1%)	446 (16.4%)
Gastrointestinal comorbid conditions					
Biliary tract disorder; n (%)	447 (4.2%)	3,409 (5.6%)	1,801 (4.5%)	39 (4.3%)	100 (3.7%)
Pancreatitis; n (%)	88 (0.8%)	714 (1.2%)	288 (0.7%)	-	15 (0.6%)
Gastroparesis; n (%)	402 (3.8%)	1,715 (2.8%)	1,261 (3.2%)	-	25 (0.9%)
MAFLD; n (%)	1,250 (11.7%)	4,000 (6.6%)	3,396 (8.6%)	97 (10.7%)	240 (8.8%)
Alcohol abuse or dependence; n (%)	189 (1.8%)	1,462 (2.4%)	739 (1.9%)	25 (2.8%)	71 (2.6%)
Other comorbid conditions					
Obstructive sleep apnea; n (%)	4,120 (38.6%)	15,395 (25.4%)	11,587 (29.3%)	348 (38.3%)	1,011 (37.2%)
Ambulatory positive pressure ventilation use; n (%)	2,241 (21.0%)	7,279 (12.0%)	6,022 (15.2%)	181 (19.9%)	547 (20.1%)
Osteoarthritis; n (%)	3,907 (36.6%)	18,901 (31.2%)	13,365 (33.8%)	425 (46.8%)	1,215 (44.7%)
Diabetes medication use					
SGLT2i; n (%)	3,580 (33.6%)	-	11,122 (28.1%)	-	-
GLP-1RA; n (%)	1,709 (16.0%)	4,951 (8.2%)	-	-	-
SGLT2i/GLP-1RA dual therapy; n (%)	2,012 (18.9%)	12,855 (21.2%)	11,122 (28.1%)	-	109 (3.4%)
Cardiovascular medication use					
ACEi/ARB/ARNI; n (%)	8,328 (78.1%)	49,101 (81.0%)	30,660 (77.4%)	551 (60.6%)	1,641 (60.4%)
β blockers; n (%)	5,481 (51.4%)	37,196 (61.4%)	20,718 (52.3%)	368 (40.5%)	1,176 (43.3%)
Calcium channel blockers; n (%)	4,220 (39.6%)	28,116 (46.4%)	16,713 (42.2%)	299 (32.9%)	935 (34.4%)
Nitrates and other antianginal agents; n (%)	940 (8.8%)	7,825 (12.9%)	3,686 (9.3%)	44 (4.8%)	150 (5.5%)
Statins; n (%)	8,853 (83.0%)	52,144 (86.1%)	33,274 (84.0%)	522 (57.4%)	1,665 (61.2%)
Antiplatelet agents; n (%)	1,279 (12.0%)	10,337 (17.1%)	5,239 (13.2%)	54 (5.9%)	174 (6.4%)
Anticoagulants (oral); n (%)	1,511 (14.2%)	13,282 (21.9%)	5,791 (14.6%)	125 (13.8%)	398 (14.6%)
Renal medication use					
Loop diuretics; n (%)	3,045 (28.6%)	23,082 (38.1%)	11,375 (28.7%)	230 (25.3%)	676 (24.9%)
Thiazide and thiazide-like diuretics; n (%)	2,288 (21.5%)	12,397 (20.5%)	8,265 (20.9%)	174 (19.1%)	536 (19.7%)
Mineralocorticoid receptor antagonists; n (%)	1,247 (11.7%)	9,589 (15.8%)	3,861 (9.8%)	86 (9.5%)	279 (10.3%)

Abbreviations: ACEi, angiotensin-converting enzyme inhibitor; ARB, angiotensin receptor blocker; ARNI, angiotensin receptor-neprilysin inhibitor; BMI, body mass index; CKD, chronic kidney disease; eGFR, estimated glomerular filtration rate; GLP-1RA, glucagon-like peptide-1 receptor antagonist; SD, standard deviation; MAFLD, metabolic dysfunction-associated fatty liver disease; SGLT2i, sodium glucose cotransporter-2 inhibitor.

a Tirzepatide and semaglutide were chosen as they are the most used anti-obesity medications.

b This GLP-1RA variable does not include semaglutide 2.4 mg SQ, and liraglutide 3 mg SQ.

c Others include Native American, Native Alaskan, Native Hawaiian.

d HbA1c was available in 61.3% (tirzepatide; CKD and T2D), 61.2% (SGLT2i), 62.3% (GLP-1RA), 46.4% (tirzepatide; CKD without diabetes), and 47% (semaglutide ≤2 mg).

e BMI and obesity information was not available in 25.5% (tirzepatide; CKD and T2D), 44.1% (SGLT2i), 35.4% (GLP-1RA), 16.8% (tirzepatide; CKD without diabetes), and 17.2% (semaglutide ≤2 mg).

f eGFR calculated from the Modification of Diet in Renal Disease (MDRD) race-free equation.

**Figure 2:**
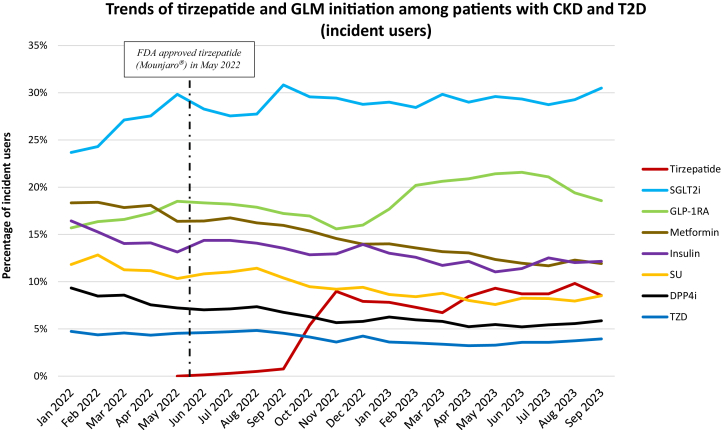
Trends of initiation of tirzepatide and glucose-lowering medications (GLMs) among patients with CKD and T2D. Denominator is patients initiated on any GLMs for a given month. CKD, chronic kidney disease; T2D, type 2 diabetes.

For incident users, tirzepatide accounted for 8.6% of all initiated GLMs by the end of September 2023. Tirzepatide initiation rapidly increased from August 2022 to December 2022 (0.5% to 7.9%) (Fig 2). Among all initiated GLMs, SGLT2i was the most frequently initiated GLM in patients with CKD and T2D (23.7% in January 2022 and 30.5% by September 2023). GLP-1RA accounted for 15%-19% of all initiated GLMs from January 202 to November 2022, then peaked at 21.6% in June 2023. There was a gradual decline in the initiation of metformin, insulin, SU, DPP4i, and TZD throughout the study period.

### CKD and T2D Cohort: Clinical Characteristics of Patients on Tirzepatide and Other GLMs

Among patients with CKD and T2D, 13,348 were initiated on tirzepatide, 84,008 were initiated on SGLT2i, and 58,705 were initiated on GLP-1RA, from January 1, 2022, to September 30, 2023 (Table 1 and Table S1). The SGLT2i initiators were older (71.6 ± 8.9 years) compared with tirzepatide (64.7 ± 9.7 years) and GLP-1RA (68.4 ± 9.1 years) initiators. Patients who were initiated on SGLT2i also had the highest combined comorbid condition score, and the highest proportion with frailty, diabetic nephropathy, diabetic retinopathy, coronary atherosclerosis, congestive heart failure, proteinuria, hyperkalemia, acute kidney injury, and lower mean eGFR compared with tirzepatide and GLP-1RA initiators. The mean eGFR for tirzepatide, SGLT2i, and GLP-1RA were 59.3 ± 20.4, 55.9 ± 19.9, and 52.3 ± 18.0 mL/min/1.73m^2^, respectively.

Patients who were initiated on GLP-1RA had the highest proportion of glycated hemoglobin level ≥ 9.0% (15.0%) compared with tirzepatide (13.2%) and SGLT2i initiators (11.7%). Tirzepatide initiators had the highest proportion of coded obesity (32.5%) and morbid obesity (44.1%), compared with GLP-1RA (29.4% for coded obesity and 32.2% for morbid obesity) and SGLT2i initiators (25.4% for coded obesity and 21.1% for morbid obesity). Patients who were initiated on tirzepatide had a higher proportion of obstructive sleep apnea and osteoarthritis compared with SGLT2i and GLP-1RA initiators. Clinical characteristics of other GLMs are depicted in Table S2.

### CKD Without Diabetes Cohort: Utilization Trends of Tirzepatide and Other AOMs

**Figure 3:**
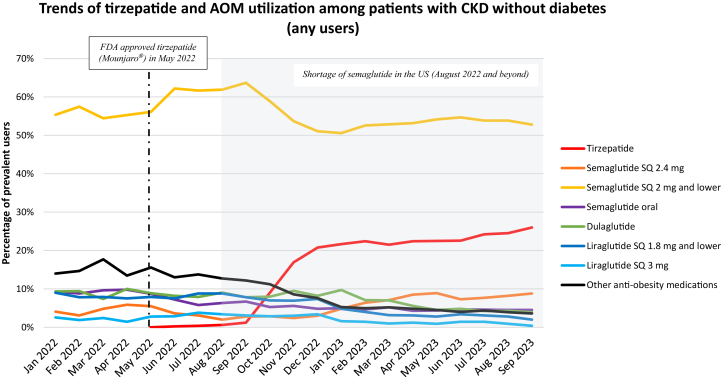
Trends of any use of tirzepatide and other anti-obesity medications (AOMs) among patients with CKD without diabetes. Denominator is patients prescribed any AOMs for a given month. Other anti-obesity medications include phentermine, naltrexone, bupropion, benzphentamine, diethylpropion, phendimetrazine, and orlistat.

We identified 5,978 patients with CKD without previous history of diabetes (Table 1 and Table S1). Among any users of AOMs in this cohort, SQ semaglutide ≤ 2 mg was the most prescribed AOM (55.4% of any users of AOMs in January 2022, peaking at 63.7% in September 2022 and decreasing to 52.8% by September 2023) (Fig 3). Tirzepatide was the second most prescribed AOM in patients with CKD without diabetes (0.2% in June 2022 and 26.0% by September 2023). The proportion of any users of SQ semaglutide 2.4 mg, oral semaglutide, dulaglutide, liraglutide, or other AOMs was each less than 10.0% after January 2023.

**Figure 4:**
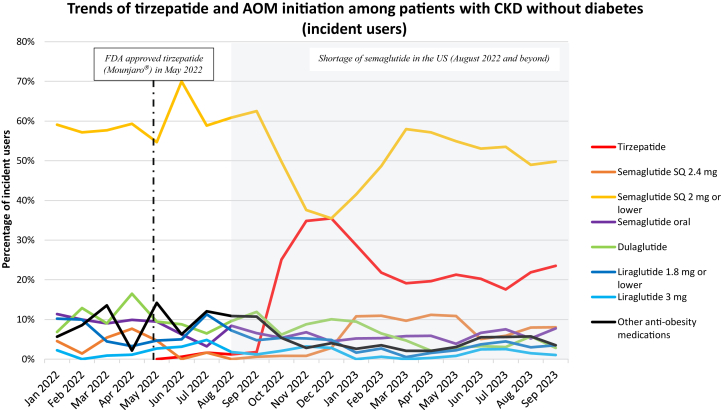
Trends of initiation of tirzepatide and anti-obesity medications (AOMs) among patients with CKD without diabetes. Denominator is patients initiated on any AOMs for a given month. Other anti-obesity medications include phentermine, naltrexone, bupropion, benzphentamine, diethylpropion, phendimetrazine, and orlistat.

Among incident users of AOMs, weekly SQ semaglutide ≤2 mg was the most frequently initiated medication throughout the study period (59.1% in January 2022 with a peak of 70.0% in June 2022) (Fig 4). Of all incident users of AOMs, incident users of tirzepatide rose from 0.6% in June 2022 to 35.5% in December 2022, then declined to 23.5% by September 2023.

### CKD without Diabetes Cohort: Clinical Characteristics of Patients on Tirzepatide and Semaglutide

Among patients with CKD without diabetes, 1,116 initiated tirzepatide and 3,200 initiated weekly SQ semaglutide ≤2 mg, from January 1, 2022, to September 30, 2023 (Table 1 and Table S1). The mean age was relatively similar among initiators of these 2 medications (64.8 ± 10.3 years for tirzepatide and 66.3 ± 9.4 years for weekly SQ semaglutide ≤2 mg). The initiators of these 2 medications had similar comorbid condition score, frailty score, prevalence of coded obesity, morbid obesity, CKD stage 3A-4, renal and cardiovascular medication use, and health care utilization (Table 1). The mean eGFR was 59.9 mL/min/1.73m^2^ in tirzepatide initiators and 58.4 mL/min/1.73m^2^ in weekly SQ semaglutide ≤2 mg initiators. Initiators of these 2 medications also had a similar proportion of acute kidney injury, biliary tract disorder, and metabolic dysfunction-associated fatty liver disease before medication initiation. Clinical characteristics of other AOMs are depicted in Table S3.

## Discussion

Our study found that tirzepatide use steadily increased since FDA approval in May 2022 among patients with CKD. There was an initial surge of any users of tirzepatide from June 2022 to December 2022, which corresponds to the reciprocal trends of GLP-1RA, possibly due to a global shortage of certain GLP-1RA, particularly semaglutide.^15^ Moreover, the rapid uptake of tirzepatide from June 2022 to December 2022 could also be driven by the swift rollout of the drug by its parent company, Eli Lilly and Company. The company reported that tirzepatide (Mounjaro) only generated $16 million of revenue in the second quarter of 2022.^16^ Then, the revenue grew substantially to $187 million in the third quarter of 2022^17^ and $279 million in the fourth quarter of 2022.^18^

By September 2023, tirzepatide users accounted for 5% of any users of GLMs, and close to 10% of incident users of GLMs among patients with CKD and T2D. In patients with CKD and T2D, tirzepatide initiators had the highest proportion of obesity and morbid obesity, obstructive sleep apnea, and osteoarthritis compared with SGLT2i and GLP-1RA initiators. These trends suggest that clinicians consider patients’ concomitant obesity status before prescribing a GLM to maximize the benefits of both glycemic control and weight management for patients with CKD and T2D.

In patients with CKD without diabetes, weekly SQ semaglutide ≤2 mg was the most frequently prescribed and initiated AOM, followed by tirzepatide during the study period. It is worth noting that our data collection ended (September 2023) before tirzepatide was officially approved for weight management by the FDA (November 2023), and thus the use of tirzepatide for weight reduction was considered off-label during our study period. Nonetheless, the uptake of tirzepatide as an AOM was rapid and large—accounting for more than one-third of all initiated AOMs in December 2022 and up to a quarter of all initiated AOMs by September 2023. The clinical characteristics were similar between tirzepatide and weekly SQ semaglutide ≤2 mg initiators, which could suggest no favorable features for physicians to prescribe one medication over the other.

Whether or not tirzepatide has kidney protective effects remains to be investigated. In 2024, the FLOW trial showed that semaglutide ≤2 mg reduced the risk of clinically important kidney outcomes and death from cardiovascular causes in patients with CKD and T2D.^19^ Whether these kidney protection and mortality benefits extend to patients who are prescribed tirzepatide or those with CKD without T2D is not fully known. Notably, the benefits of tirzepatide on kidney outcomes were observed in at least one study. In a post hoc analysis of the SURPASS-4 trial, tirzepatide was associated with a slower eGFR decline but unchanged urine albumin:creatinine ratio compared with insulin glargine, among patients with CKD.^20^ The effect on eGFR change was most prominent in patients with early CKD (eGFR ≥90 mL/min/1.73m^2^). However, there were limitations to this post hoc analysis. The proportion of patients with baseline CKD in the study, defined as eGFR < 60 mL/min/1.73m^2^, was only 18%, and CKD stages were not clearly classified. These limitations present a need for future comparative effectiveness and safety studies of tirzepatide against other medications in patients with CKD irrespective of T2D status. Interestingly, one retrospective cohort study of 14,834 patients with T2D suggests that tirzepatide may portray more cardiovascular and renal benefits than a GLP-1RA.^21^ A randomized controlled trial, the TREASURE-CKD trial (NCT05536804), is currently enrolling participants to evaluate renal outcomes of tirzepatide versus placebo in adults with CKD who are overweight or obese, with or without T2D.

Among the GLMs, SGLT2i was the most frequently initiated GLM (∼30%) for patients with CKD and T2D in our study. Patients who were initiated on SGLT2i had a higher comorbid condition burden, prevalence of cardiovascular disease, and kidney-related complications, compared with tirzepatide and GLP-1RA initiators. Our study found that the utilization of SGLT2i among patients with CKD and T2D has continued to increase beyond what has been previously reported,^14^^,^^22^ likely related to the publication of several landmark clinical trials demonstrating cardiovascular and renoprotective benefits in patients with CKD and T2D.^11^^,^^23^, ^24^, ^25^, ^26^, ^27^ Accumulating evidence also has promoted the Kidney Disease Improving Global Outcomes (KDIGO) guidelines recommending SGLT2i and, to a lesser extent, GLP-1RA for patients with CKD and T2D.^28^

Our study has some limitations. First, the study period only extended to September 2023, before the FDA approval of tirzepatide for weight management. Second, we had limited data available on laboratory results, such as the degree of proteinuria, albuminuria, and eGFR. Third, obesity-related diagnoses are generally undercoded and direct body mass index (BMI) measurement is not available from claims databases. However, obesity-related codes were validated and have high specificity (99.7% specificity for obesity, 97.4% specificity for overweight).^29^ Fourth, we used a claims-based definition of CKD, which may not capture all patients with CKD in the population. Nonetheless, our CKD claims codes were validated with high positive predictive values and high specificity.^10^

In conclusion, our study indicates rapidly shifting trends in the uptake of tirzepatide in patients with CKD both with and without diabetes. In patients with CKD and T2D, tirzepatide initiation rapidly rose over time, whereas SGLT2i still remained the most initiated GLM. In patients with CKD without diabetes, weekly SQ semaglutide ≤2 mg and tirzepatide were the 2 most frequently initiated AOMs. The uptake of tirzepatide initiation in patients with CKD without diabetes was rapid and sustained. Future studies on the comparative effectiveness and safety of tirzepatide against other GLMs or AOMs in patients with CKD are needed.
