# Supplementary material for: Utilization Trends of Dual GIP/GLP-1 Receptor Agonist, Newer Glucose-Lowering Medications, and Anti-Obesity Medications Among Patients With Chronic Kidney Disease With and Without Type 2 Diabetes
Source: Kidney Med. 2025 Apr 19;7(6):101013. doi: 10.1016/j.xkme.2025.101013 (PMC12152630; doi:10.1016/j.xkme.2025.101013)
Supplement: Supplementary File (PDF) — Tables S1-S3. [file mmc1.pdf]

Table *S*1. Summary of cohort inclusions and exclusions for **A)** CKD and T2D cohort and **B)** CKD without diabetes cohort.

Cohort entry date: January 1, 2022 to September 30, 2023
Cohort entry definition *(CKD and T2D): prescriptions for any glucose-lowering medications (GLM)*
Cohort entry definition *(CKD without diabetes): prescriptions for any anti-obesity medications (AOM)*
Database: Optum's de-identified Clinformatics® Data Mart Database

**A) CKD and T2D Cohort**	**Change**	**Value(n)**
Patients in Optum's de-identified Clinformatics® Data Mart Database		90,752,871
Patients meeting cohort entry criteria (prescribed at least one GLM)		2,860,765
Excluded due to insufficient enrollment (<365 days)	-778,571 (27%)	2,082,194
Excluded patients without CKD or ESKD	-1,400,145 (67%)	682,049
Excluded patients without T2D	-25,505 (4%)	656,544
Excluded patients with missing gender variable	-41 (<1%)	656,503
Excluded patients with missing age variable	-17 (<1%)	656,486
Excluded patients aged less than18 years	-186 (<1%)	656,300
Excluded patients with type 1 diabetes	-120,278 (18%)	536,022
Excluded patients with secondary and gestational diabetes	-80,975 (15%)	455,047
Total Patients		455,047

**B) CKD without diabetes Cohort**	**Change**	**Value(n)**
Patients in Optum's de-identified Clinformatics® Data Mart Database		90,752,871
Patients meeting cohort entry criteria (prescribed at least one AOM)		784,071
Excluded due to insufficient enrollment (< 365 days)	-224,897 (29%)	559,174
Excluded patients without CKD or ESKD	-406,145 (73%)	153,029
Excluded patients with missing age variable	-1 (<1%)	153,028
Excluded patients aged less than l8 years	-0 (<1%)	153,028
Excluded patients without T2D	-146,888 (96%)	6,140
Excluded patients with type 1 diabetes	-39 (<1%)	6,101
Excluded patients who were taking T2D-specific drugs	-67 (1%)	6,034
Excluded patients with secondary and gestational diabetes	-55 (<1%)	5,979
Excluded patients with missing gender variable	-1 (<1%)	5,978
Total Patients		5,978

CKD, chronic kidney disease; DM, diabetes mellitus; ESKD, end-stage kidney disease; T2D, type 2 diabetes

Table S2. Clinical characteristics of initiators of tirzepatide and glucose-lowering medications among patients with CKD and T2D from January 1, 2022 to September 30, 2023.

	Tirzepatide	SGLT2i	GLP-1RA*	Insulin	Metformin	SU	DPP4i	TZD
Number of patients	13,348	84,008	58,705	47,600	69,168	39,887	23,213	14,234
**Demographics**								
Mean age, years (SD)	64.71 (9.68)	71.60 (8.92)	68.41 (9.11)	70.83 (9.74)	70.96 (9.52)	71.93 (9.08)	73.14 (8.93)	71.68 (8.50)
Gender - female; n (%)	7,733 (57.9%)	38,464 (45.8%)	32,468 (55.3%)	23,263 (48.9%)	33,739 (48.8%)	19,071 (47.8%)	12,117 (52.2%)	6,760 (47.5%)
Race categories								
...White; n (%)	8,532 (63.9%)	47,879 (57.0%)	35,052 (59.7%)	27,413 (57.6%)	39,900 (57.7%)	22,903 (57.4%)	12,335 (53.1%)	7,773 (54.6%)
...Black; n (%)	2,095 (15.7%)	13,638 (16.2%)	8,988 (15.3%)	7,400 (15.5%)	9,783 (14.1%)	5,703 (14.3%)	3,680 (15.9%)	1,645 (11.6%)
...Asian; n (%)	204 (1.5%)	3,267 (3.9%)	1,344 (2.3%)	1,182 (2.5%)	2,481 (3.6%)	1,384 (3.5%)	964 (4.2%)	607 (4.3%)
...Hispanic; n (%)	1,491 (11.2%)	11,173 (13.3%)	7,441 (12.7%)	5,843 (12.3%)	9,125 (13.2%)	5,285 (13.2%)	3,542 (15.3%)	2,609 (18.3%)
...Others; n (%)[†]	1,026 (7.7%)	8,051 (9.6%)	5,880 (10.0%)	5,762 (12.1%)	7,879 (11.4%)	4,612 (11.6%)	2,692 (11.6%)	1,600 (11.2%)
Mean combined comorbidity score (SD)	3.51 (2.81)	4.33 (3.31)	3.77 (2.99)	5.01 (3.57)	3.75 (3.19)	3.93 (3.19)	4.18 (3.33)	3.35 (2.85)
Frailty Score								
...0.00 - 0.14 (robust); n (%)	256 (1.9%)	1,663 (2.0%)	1,109 (1.9%)	596 (1.3%)	1,852 (2.7%)	886 (2.2%)	456 (2.0%)	436 (3.1%)
...0.15 - 0.24 (pre-frail); n (%)	8,540 (64.0%)	51,591 (61.4%)	37,117 (63.2%)	24,876 (52.3%)	44,541 (64.4%)	25,655 (64.3%)	14,109 (60.8%)	10,098 (70.9%)
...≥ 0.25 (frail); n (%)	4,552 (34.1%)	30,754 (36.6%)	20,479 (34.9%)	22,128 (46.5%)	22,775 (32.9%)	13,346 (33.5%)	8,648 (37.3%)	3,700 (26.0%)
**Diabetes comorbidities**								
Diabetic nephropathy; n (%)	7,902 (59.2%)	54,049 (64.3%)	37,737 (64.3%)	34,522 (72.5%)	40,718 (58.9%)	26,762 (67.1%)	15,664 (67.5%)	9,685 (68.0%)
Diabetic neuropathy; n (%)	4,547 (34.1%)	26,759 (31.9%)	20,938 (35.7%)	20,210 (42.5%)	19,329 (27.9%)	13,414 (33.6%)	7,969 (34.3%)	4,888 (34.3%)
Diabetic retinopathy; n (%)	1,851 (13.9%)	12,798 (15.2%)	9,374 (16.0%)	10,168 (21.4%)	7,618 (11.0%)	5,676 (14.2%)	3,594 (15.5%)	2,178 (15.3%)
Diabetic ketoacidosis; n (%)	75 (0.6%)	504 (0.6%)	435 (0.7%)	899 (1.9%)	507 (0.7%)	284 (0.7%)	185 (0.8%)	81 (0.6%)
Hypoglycemia; n (%)	529 (4.0%)	3,746 (4.5%)	2,849 (4.9%)	4,029 (8.5%)	2,393 (3.5%)	1,834 (4.6%)	1,159 (5.0%)	610 (4.3%)
Mean HbA1c; % (SD)[‡]	7.41 (1.50)	7.40 (1.48)	7.60 (1.56)	7.99 (1.70)	7.28 (1.46)	7.63 (1.48)	7.58 (1.44)	7.76 (1.49)
HbA1c < 7.5%; n (%)	5,802 (43.5%)	35,650 (42.4%)	23,330 (39.7%)	14,991 (31.5%)	30,055 (43.5%)	15,506 (38.9%)	9,177 (39.5%)	5,626 (39.5%)
HbA1c 7.5-8.9%; n (%)	3,024 (22.7%)	19,173 (22.8%)	14,906 (25.4%)	12,411 (26.1%)	12,291 (17.8%)	9,968 (25.0%)	5,894 (25.4%)	4,281 (30.1%)
HbA1c ≥ 9.0%; n (%)	1,758 (13.2%)	9,852 (11.7%)	8,783 (15.0%)	8,715 (18.3%)	6,701 (9.7%)	5,337 (13.4%)	3,027 (13.0%)	2,372 (16.7%)
**Metabolic comorbidities[§]**								
Underweight or Normal Weight (BMI <25 kg/m^2); n (%)	151 (1.1%)	4,005 (4.8%)	1,337 (2.3%)	2,500 (5.3%)	3,223 (4.7%)	1,917 (4.8%)	1,360 (5.9%)	758 (5.3%)

Overweight (BMI 25-29.9 kg/m^2); n (%)	1,148 (8.6%)	11,799 (14.0%)	6,615 (11.3%)	6,100 (12.8%)	9,204 (13.3%)	5,371 (13.5%)	3,413 (14.7%)	2,015 (14.2%)
Obese (BMI 30-39.9 kg/m^2); n (%)	4,341 (32.5%)	21,367 (25.4%)	17,276 (29.4%)	11,955 (25.1%)	16,917 (24.5%)	9,130 (22.9%)	5,073 (21.9%)	3,267 (23.0%)
Morbid obese (BMI ≥ 40 kg/m^2); n (%)	5,890 (44.1%)	17,697 (21.1%)	18,882 (32.2%)	10,853 (22.8%)	14,322 (20.7%)	7,387 (18.5%)	3,669 (15.8%)	2,491 (17.5%)
**Cardiovascular comorbidities**								
Hypertension; n (%)	12,207 (91.5%)	78,571 (93.5%)	53,727 (91.5%)	43,860 (92.1%)	60,889 (88.0%)	35,899 (90.0%)	21,286 (91.7%)	12,698 (89.2%)
Hyperlipidemia; n (%)	11,631 (87.1%)	73,559 (87.6%)	50,800 (86.5%)	40,917 (86.0%)	58,508 (84.6%)	34,035 (85.3%)	20,080 (86.5%)	12,425 (87.3%)
Gout; n (%)	1,464 (11.0%)	10,299 (12.3%)	6,127 (10.4%)	4,934 (10.4%)	6,378 (9.2%)	3,978 (10.0%)	2,251 (9.7%)	1,181 (8.3%)
Coronary atherosclerosis; n (%)	3,493 (26.2%)	31,716 (37.8%)	17,072 (29.1%)	17,614 (37.0%)	19,418 (28.1%)	11,837 (29.7%)	7,182 (30.9%)	3,281 (23.1%)
Congestive heart failure; n (%)	2,698 (20.2%)	28,292 (33.7%)	13,106 (22.3%)	15,319 (32.2%)	14,244 (20.6%)	9,078 (22.8%)	5,474 (23.6%)	1,944 (13.7%)
Atrial fibrillation; n (%)	1,618 (12.1%)	18,353 (21.8%)	8,211 (14.0%)	9,490 (19.9%)	10,614 (15.3%)	6,434 (16.1%)	3,897 (16.8%)	1,467 (10.3%)
Ischemic stroke; n (%)	1,254 (9.4%)	11,772 (14.0%)	6,692 (11.4%)	7,769 (16.3%)	8,439 (12.2%)	5,067 (12.7%)	3,196 (13.8%)	1,462 (10.3%)
Peripheral arterial disease; n (%)	1,617 (12.1%)	14,620 (17.4%)	8,704 (14.8%)	9,421 (19.8%)	10,583 (15.3%)	6,387 (16.0%)	4,053 (17.5%)	2,159 (15.2%)
Smoking; n (%)	3,993 (29.9%)	26,823 (31.9%)	17,828 (30.4%)	16,330 (34.3%)	21,054 (30.4%)	11,627 (29.1%)	6,601 (28.4%)	3,576 (25.1%)
**Cholesterol panel**‡								
Mean HDL cholesterol (mg/dL) (SD)	54.59 (55.31)	54.68 (50.31)	54.20 (50.64)	52.17 (42.46)	52.81 (40.28)	52.74 (45.40)	54.62 (51.41)	56.46 (66.04)
Mean LDL cholesterol (mg/dL) (SD)	108.22 (120.62)	101.90 (101.39)	148.85 (7,602.90)	249.57 (21,596.22)	196.67 (17,134.64)	264.31 (22,494.74)	101.58 (99.55)	106.51 (137.04)
Mean Triglycerides (mg/dL) (SD)	216.57 (275.57)	194.39 (234.49)	208.51 (240.78)	206.50 (247.59)	188.64 (216.59)	198.90 (218.86)	194.42 (209.05)	209.01 (271.79)
**Renal comorbidities**								
CKD stage 1-2; n (%)	3,110 (23.3%)	16,448 (19.6%)	12,242 (20.9%)	8,051 (16.9%)	16,180 (23.4%)	7,835 (19.6%)	4,316 (18.6%)	3,315 (23.3%)
CKD stage 3a-4; n (%)	7,447 (55.8%)	56,072 (66.7%)	35,561 (60.6%)	31,045 (65.2%)	39,055 (56.5%)	25,137 (63.0%)	14,821 (63.8%)	8,563 (60.2%)
CKD stage 5 and dialysis; n (%)	340 (2.5%)	1,635 (1.9%)	1,935 (3.3%)	3,920 (8.2%)	1,078 (1.6%)	1,441 (3.6%)	1,087 (4.7%)	337 (2.4%)
CKD unspecified; n (%)	3,125 (23.4%)	23,088 (27.5%)	14,470 (24.6%)	15,451 (32.5%)	15,149 (21.9%)	9,902 (24.8%)	6,274 (27.0%)	2,844 (20.0%)
Proteinuria; n (%)	1,642 (12.3%)	12,799 (15.2%)	7,532 (12.8%)	6,745 (14.2%)	6,753 (9.8%)	4,758 (11.9%)	2,793 (12.0%)	1,562 (11.0%)
Hyperkalemia; n (%)	856 (6.4%)	8,242 (9.8%)	4,829 (8.2%)	6,588 (13.8%)	4,655 (6.7%)	3,626 (9.1%)	2,434 (10.5%)	1,033 (7.3%)
Acute kidney injury; n (%)	1,969 (14.8%)	18,257 (21.7%)	10,019 (17.1%)	13,354 (28.1%)	11,308 (16.3%)	7,650 (19.2%)	4,857 (20.9%)	1,961 (13.8%)
Mean eGFR** (mL/min/1.73m^2) (SD)‡	59.28 (20.37)	52.32 (18.04)	55.85 (19.91)	50.40 (21.01)	59.38 (17.97)	53.63 (19.53)	51.76 (19.54)	56.16 (19.53)
Urinary tract infection; n (%)	2,328 (17.4%)	13,851 (16.5%)	10,455 (17.8%)	10,693 (22.5%)	11,904 (17.2%)	7,030 (17.6%)	4,750 (20.5%)	2,287 (16.1%)
**Gastrointestinal comorbidities**								
Biliary tract disorder; n (%)	574 (4.3%)	4,745 (5.6%)	2,900 (4.9%)	3,520 (7.4%)	3,546 (5.1%)	2,150 (5.4%)	1,278 (5.5%)	674 (4.7%)
Pancreatitis; n (%)	125 (0.9%)	990 (1.2%)	533 (0.9%)	967 (2.0%)	828 (1.2%)	478 (1.2%)	246 (1.1%)	166 (1.2%)
Gastroparesis; n (%)	489 (3.7%)	2,448 (2.9%)	2,004 (3.4%)	2,004 (4.2%)	1,752 (2.5%)	1,066 (2.7%)	721 (3.1%)	406 (2.9%)

MAFLD; n (%)	1,564 (11.7%)	5,754 (6.8%)	5,245 (8.9%)	3,660 (7.7%)	5,074 (7.3%)	2,653 (6.7%)	1,455 (6.3%)	943 (6.6%)
Alcohol abuse or dependence; n (%)	257 (1.9%)	2,008 (2.4%)	1,155 (2.0%)	1,391 (2.9%)	1,878 (2.7%)	883 (2.2%)	452 (1.9%)	261 (1.8%)
**Other comorbidities**								
Obstructive sleep apnea; n (%)	5,108 (38.3%)	21,159 (25.2%)	17,291 (29.5%)	11,669 (24.5%)	15,146 (21.9%)	7,753 (19.4%)	4,242 (18.3%)	2,404 (16.9%)
Ambulatory positive pressure ventilation use; n (%)	2,722 (20.4%)	10,129 (12.1%)	8,771 (14.9%)	4,917 (10.3%)	6,954 (10.1%)	3,522 (8.8%)	1,930 (8.3%)	1,125 (7.9%)
Osteoarthritis; n (%)	4,831 (36.2%)	26,150 (31.1%)	19,821 (33.8%)	15,262 (32.1%)	21,350 (30.9%)	11,796 (29.6%)	7,335 (31.6%)	3,881 (27.3%)
**Diabetes medication use**								
SGLT2i; n (%)	3,814 (28.6%)	42,518 (50.6%)	15,176 (25.9%)	11,225 (23.6%)	12,744 (18.4%)	8,636 (21.7%)	5,275 (22.7%)	3,615 (25.4%)
GLP-1RA; n (%)	3,200 (24.0%)	11,043 (13.1%)	26,864 (45.8%)	9,354 (19.7%)	9,198 (13.3%)	5,491 (13.8%)	2,431 (10.5%)	2,183 (15.3%)
SGLT2i/GLP-1RA dual therapy; n (%)	2,354 (17.6%)	10,147 (12.1%)	14,758 (25.1%)	5,244 (11.0%)	4,569 (6.6%)	3,090 (7.7%)	1,560 (6.7%)	1,529 (10.7%)
**Cardiovascular medication use**								
ACEi/ARB/ARNI; n (%)	10,402 (77.9%)	67,862 (80.8%)	45,552 (77.6%)	34,651 (72.8%)	51,367 (74.3%)	30,347 (76.1%)	17,493 (75.4%)	11,275 (79.2%)
Beta blockers; n (%)	6,721 (50.4%)	50,003 (59.5%)	30,531 (52.0%)	27,411 (57.6%)	32,953 (47.6%)	20,563 (51.6%)	12,265 (52.8%)	6,321 (44.4%)
Calcium channel blockers; n (%)	5,126 (38.4%)	37,291 (44.4%)	24,188 (41.2%)	21,407 (45.0%)	27,707 (40.1%)	17,062 (42.8%)	10,404 (44.8%)	5,682 (39.9%)
Nitrates and other antianginal agents; n (%)	1,137 (8.5%)	10,340 (12.3%)	5,555 (9.5%)	5,808 (12.2%)	5,348 (7.7%)	3,641 (9.1%)	2,174 (9.4%)	853 (6.0%)
Statins; n (%)	10,850 (81.3%)	71,652 (85.3%)	49,051 (83.6%)	38,981 (81.9%)	55,710 (80.5%)	32,652 (81.9%)	19,323 (83.2%)	12,121 (85.2%)
Antiplatelet agents; n (%)	1,628 (12.2%)	14,049 (16.7%)	7,844 (13.4%)	8,442 (17.7%)	8,738 (12.6%)	5,711 (14.3%)	3,418 (14.7%)	1,586 (11.1%)
Anticoagulants (oral); n (%)	1,798 (13.5%)	17,436 (20.8%)	8,540 (14.5%)	9,014 (18.9%)	10,397 (15.0%)	6,016 (15.1%)	3,715 (16.0%)	1,452 (10.2%)
**Renal medication use**								
Loop diuretics; n (%)	3,720 (27.9%)	29,845 (35.5%)	16,585 (28.3%)	17,086 (35.9%)	15,480 (22.4%)	10,330 (25.9%)	6,291 (27.1%)	2,556 (18.0%)
Thiazide and thiazide-like diuretics; n (%)	2,931 (22.0%)	16,924 (20.1%)	12,348 (21.0%)	8,814 (18.5%)	12,916 (18.7%)	7,553 (18.9%)	4,335 (18.7%)	2,611 (18.3%)
Mineralocorticoid receptor antagonists; n (%)	1,476 (11.1%)	12,583 (15.0%)	5,833 (9.9%)	4,958 (10.4%)	5,699 (8.2%)	3,171 (7.9%)	1,784 (7.7%)	795 (5.6%)
**Healthcare utilization**								
ED event; n (%)	3,790 (28.4%)	30,610 (36.4%)	19,428 (33.1%)	21,135 (44.4%)	23,112 (33.4%)	14,019 (35.1%)	8,868 (38.2%)	4,243 (29.8%)
Hospitalization event; n (%)	2,436 (18.2%)	21,718 (25.9%)	11,959 (20.4%)	16,422 (34.5%)	15,298 (22.1%)	9,164 (23.0%)	5,724 (24.7%)	2,362 (16.6%)
Internist; n (%)	12,651 (94.8%)	80,179 (95.4%)	55,899 (95.2%)	45,101 (94.8%)	65,221 (94.3%)	37,670 (94.4%)	22,069 (95.1%)	13,482 (94.7%)
Nephrologist; n (%)	3,043 (22.8%)	22,594 (26.9%)	13,582 (23.1%)	14,204 (29.8%)	11,123 (16.1%)	8,867 (22.2%)	5,927 (25.5%)	2,542 (17.9%)
Endocrinologist; n (%)	2,639 (19.8%)	11,019 (13.1%)	9,529 (16.2%)	8,840 (18.6%)	6,870 (9.9%)	4,266 (10.7%)	2,811 (12.1%)	1,577 (11.1%)
Cardiologist; n (%)	6,184 (46.3%)	43,918 (52.3%)	26,764 (45.6%)	25,033 (52.6%)	30,102 (43.5%)	17,512 (43.9%)	10,847 (46.7%)	4,943 (34.7%)
Nutritionist visit; n (%)	1,145 (8.6%)	5,351 (6.4%)	4,500 (7.7%)	3,126 (6.6%)	3,844 (5.6%)	2,127 (5.3%)	1,306 (5.6%)	749 (5.3%)

ACEi, angiotensin-converting enzyme inhibitor; ARB, angiotensin receptor blocker; ARNI, angiotensin receptor-neprilysin inhibitor; BMI, body mass index; CKD, chronic kidney disease; DPP4i, dipeptidyl peptidase-4 inhibitor; ED, emergency department; eGFR, estimated glomerular filtration rate; GLP-1RA, glucagon-like peptide-1 receptor antagonist; SD, standard deviation; MAFLD, metabolic dysfunction-associated fatty liver disease; SGLT2i, sodium glucose cotransporter-2 inhibitor; SU, sulfonylurea; TZD, thiazolidinedione

†Others include Native American, Native Alaskan, Native Hawaiian

‡obtained only from patients with available lab values

§BMI and obesity information was not available in all patients

*This GLP-1RA variable does not include semaglutide 2.4 mg SQ or liraglutide 3 mg SQ

**eGFR calculated from the Modification of Diet in Renal Disease (MDRD) race-free equation

Table S3. Clinical characteristics of initiators of tirzepatide and anti-obesity medications among patients with CKD without diabetes from January 1, 2022 to September 30, 2023.

Variable	Tirzepatide	Semaglutide ≤2 mg	Semaglutide 2.4 mg SQ	Semaglutide oral	Liraglutide 3 mg	Dulaglutide	Other anti-obesity medications[#]
Number of patients	1,116	3,200	378	376	96	375	319
**Demographics**							
Mean age, years (SD)	64.79 (10.30)	66.31 (9.39)	52.85 (9.24)	68.05 (9.37)	51.74 (10.13)	67.05 (9.26)	63.24 (11.45)
Gender - female; n (%)	841 (75.4%)	2,407 (75.2%)	258 (68.3%)	245 (65.2%)	80 (83.3%)	290 (77.3%)	242 (75.9%)
Race categories							
...White; n (%)	810 (72.6%)	2,257 (70.5%)	278 (73.5%)	268 (71.3%)	70 (72.9%)	256 (68.3%)	250 (78.4%)
...Black; n (%)	129 (11.6%)	434 (13.6%)	55 (14.6%)	54 (14.4%)	15 (15.6%)	51 (13.6%)	35 (11.0%)
...Asian; n (%)	-	38 (1.2%)	-	-	-	-	-
...Hispanic; n (%)	97 (8.7%)	251 (7.8%)	20 (5.3%)	20 (5.3%)	4 (4.2%)	31 (8.3%)	20 (6.3%)
...Others; n (%)[†]	76 (6.8%)	220 (6.9%)	20 (5.3%)	28 (7.4%)	6 (6.3%)	33 (8.8%)	11 (3.4%)
Mean combined comorbidity score (SD)	2.32 (2.45)	2.42 (2.56)	1.65 (1.94)	2.21 (2.45)	1.99 (2.43)	2.55 (2.72)	1.70 (2.23)
Frailty Score							
...0.00 - 0.14 (robust); n (%)	34 (3.0%)	101 (3.2%)	30 (7.9%)	22 (5.9%)	-	-	29 (9.1%)
...0.15 - 0.24 (pre-frail); n (%)	769 (68.9%)	2,217 (69.3%)	305 (80.7%)	287 (76.3%)	76 (79.2%)	261 (69.6%)	234 (73.4%)
...≥ 0.25 (frail); n (%)	313 (28.0%)	882 (27.6%)	43 (11.4%)	67 (17.8%)	12 (12.5%)	107 (28.5%)	56 (17.6%)
**Metabolic comorbidities**[§]							
Overweight (BMI 25-29.9 kg/m^2); n (%)	127 (11.4%)	336 (10.5%)	41 (10.8%)	31 (8.2%)	18 (18.8%)	47 (12.5%)	47 (14.7%)
Obese (BMI 30-39.9 kg/m^2); n (%)	422 (37.8%)	1,185 (37.0%)	160 (42.3%)	144 (38.3%)	39 (40.6%)	129 (34.4%)	127 (39.8%)
Morbid obese (BMI ≥ 40 kg/m^2); n (%)	541 (48.5%)	1,617 (50.5%)	196 (51.9%)	161 (42.8%)	50 (52.1%)	191 (50.9%)	110 (34.5%)
**Cardiovascular comorbidities**							
Hypertension; n (%)	906 (81.2%)	2,647 (82.7%)	242 (64.0%)	301 (80.1%)	64 (66.7%)	310 (82.7%)	215 (67.4%)
Hyperlipidemia; n (%)	830 (74.4%)	2,363 (73.8%)	214 (56.6%)	295 (78.5%)	45 (46.9%)	281 (74.9%)	198 (62.1%)
Gout; n (%)	101 (9.1%)	278 (8.7%)	23 (6.1%)	28 (7.4%)	-	36 (9.6%)	19 (6.0%)
Coronary atherosclerosis; n (%)	212 (19.0%)	636 (19.9%)	33 (8.7%)	56 (14.9%)	-	62 (16.5%)	35 (11.0%)
Congestive heart failure; n (%)	159 (14.2%)	501 (15.7%)	27 (7.1%)	48 (12.8%)	-	59 (15.7%)	19 (6.0%)
Atrial fibrillation; n (%)	128 (11.5%)	376 (11.8%)	19 (5.0%)	49 (13.0%)	-	48 (12.8%)	20 (6.3%)
Ischemic stroke; n (%)	91 (8.2%)	210 (6.6%)	-	29 (7.7%)	-	21 (5.6%)	18 (5.6%)

Peripheral arterial disease; n (%)	106 (9.5%)	299 (9.3%)	-	38 (10.1%)	-	39 (10.4%)	16 (5.0%)
Smoking; n (%)	359 (32.2%)	1,076 (33.6%)	99 (26.2%)	97 (25.8%)	25 (26.0%)	152 (40.5%)	80 (25.1%)
**Cholesterol panel**[‡]							
Mean HDL cholesterol (mg/dL) (SD)	59.88 (42.69)	61.68 (59.31)	54.90 (24.35)	60.94 (69.05)	58.43 (16.61)	61.66 (44.37)	60.77 (21.20)
Mean LDL cholesterol (mg/dL) (SD)	123.61 (87.71)	123.30 (145.10)	120.20 (56.92)	125.81 (142.15)	120.13 (50.55)	122.02 (82.59)	129.72 (65.69)
Mean triglycerides (mg/dL) (SD)	153.29 (104.20)	166.79 (174.79)	151.23 (96.67)	165.43 (146.24)	133.90 (66.47)	172.96 (139.56)	141.40 (77.98)
**Renal comorbidities**							
CKD stage 1-2; n (%)	228 (20.4%)	562 (17.6%)	76 (20.1%)	81 (21.5%)	13 (13.5%)	56 (14.9%)	55 (17.2%)
CKD stage 3a-4; n (%)	607 (54.4%)	1,797 (56.2%)	156 (41.3%)	225 (59.8%)	41 (42.7%)	234 (62.4%)	140 (43.9%)
CKD stage 5 and dialysis; n (%)	12 (1.1%)	52 (1.6%)	-	-	-	-	-
CKD unspecified; n (%)	233 (20.9%)	637 (19.9%)	61 (16.1%)	62 (16.5%)	17 (17.7%)	83 (22.1%)	54 (16.9%)
Proteinuria; n (%)	62 (5.6%)	190 (5.9%)	30 (7.9%)	24 (6.4%)	-	19 (5.1%)	18 (5.6%)
Hyperkalemia; n (%)	35 (3.1%)	110 (3.4%)	-	14 (3.7%)	-	-	-
Acute kidney injury; n (%)	112 (10.0%)	301 (9.4%)	28 (7.4%)	38 (10.1%)	-	37 (9.9%)	26 (8.2%)
Mean eGFR (mL/min/1.73m^2) (SD)[‡,*]	59.93 (16.39)	58.37 (16.89)	67.70 (18.96)	57.52 (16.91)	69.01 (22.26)	56.97 (17.03)	62.12 (16.59)
Urinary tract infection; n (%)	198 (17.7%)	525 (16.4%)	37 (9.8%)	48 (12.8%)	-	55 (14.7%)	53 (16.6%)
**Gastrointestinal comorbidities**							
Biliary tract disorder; n (%)	49 (4.4%)	119 (3.7%)	14 (3.7%)	-	-	17 (4.5%)	-
Pancreatitis; n (%)	5 (0.4%)	16 (0.5%)	-	-	-	-	-
Gastroparesis; n (%)	12 (1.1%)	32 (1.0%)	-	-	-	-	-
MAFLD; n (%)	114 (10.2%)	288 (9.0%)	37 (9.8%)	37 (9.8%)	10 (10.4%)	30 (8.0%)	29 (9.1%)
Alcohol abuse or dependence; n (%)	29 (2.6%)	85 (2.7%)	-	-	-	-	-
**Other comorbidities**							
Obstructive sleep apnea; n (%)	410 (36.7%)	1,207 (37.7%)	129 (34.1%)	133 (35.4%)	30 (31.3%)	126 (33.6%)	94 (29.5%)
Ambulatory positive pressure ventilation use; n (%)	206 (18.5%)	651 (20.3%)	70 (18.5%)	77 (20.5%)	15 (15.6%)	58 (15.5%)	52 (16.3%)
Osteoarthritis; n (%)	514 (46.1%)	1,434 (44.8%)	110 (29.1%)	157 (41.8%)	25 (26.0%)	176 (46.9%)	134 (42.0%)
**Cardiovascular medication use**							
ACEi/ARB/ARNI; n (%)	655 (58.7%)	1,906 (59.6%)	178 (47.1%)	223 (59.3%)	32 (33.3%)	222 (59.2%)	149 (46.7%)
Beta blockers; n (%)	448 (40.1%)	1,371 (42.8%)	117 (31.0%)	148 (39.4%)	35 (36.5%)	173 (46.1%)	92 (28.8%)
Calcium channel blockers; n (%)	364 (32.6%)	1,088 (34.0%)	100 (26.5%)	137 (36.4%)	23 (24.0%)	131 (34.9%)	81 (25.4%)
Statins; n (%)	625 (56.0%)	1,938 (60.6%)	128 (33.9%)	238 (63.3%)	34 (35.4%)	236 (62.9%)	133 (41.7%)

Antiplatelet agents; n (%)	70 (6.3%)	196 (6.1%)	22 (5.8%)	17 (4.5%)	-	20 (5.3%)	-
**Renal medication use**							
Loop diuretics; n (%)	284 (25.4%)	782 (24.4%)	53 (14.0%)	89 (23.7%)	14 (14.6%)	105 (28.0%)	37 (11.6%)
Thiazide and thiazide-like diuretics; n (%)	212 (19.0%)	644 (20.1%)	63 (16.7%)	60 (16.0%)	15 (15.6%)	88 (23.5%)	50 (15.7%)
Mineralocorticoid receptor antagonists; n (%)	114 (10.2%)	328 (10.3%)	35 (9.3%)	25 (6.6%)	-	40 (10.7%)	29 (9.1%)
**Healthcare utilization**							
ED event; n (%)	312 (28.0%)	933 (29.2%)	12 (3.2%)	81 (21.5%)	-	116 (30.9%)	42 (13.2%)
Hospitalization event; n (%)	142 (12.7%)	411 (12.8%)	30 (7.9%)	36 (9.6%)	-	41 (10.9%)	31 (9.7%)
Internist; n (%)	1,061 (95.1%)	3,049 (95.3%)	345 (91.3%)	351 (93.4%)	89 (92.7%)	356 (94.9%)	301 (94.4%)
Nephrologist; n (%)	207 (18.5%)	584 (18.3%)	80 (21.2%)	79 (21.0%)	20 (20.8%)	69 (18.4%)	55 (17.2%)
Endocrinologist; n (%)	91 (8.2%)	282 (8.8%)	58 (15.3%)	30 (8.0%)	17 (17.7%)	34 (9.1%)	31 (9.7%)
Cardiologist; n (%)	477 (42.7%)	1,369 (42.8%)	124 (32.8%)	144 (38.3%)	33 (34.4%)	139 (37.1%)	108 (33.9%)
Nutritionist visit; n (%)	89 (8.0%)	315 (9.8%)	77 (20.4%)	35 (9.3%)	22 (22.9%)	38 (10.1%)	36 (11.3%)

ACEi, angiotensin-converting enzyme inhibitor; ARB, angiotensin receptor blocker; ARNI, angiotensin receptor-neprilysin inhibitor; BMI, body mass index; CKD, chronic kidney disease; DPP4i, dipeptidyl peptidase-4 inhibitor; ED, emergency department; eGFR, estimated glomerular filtration rate; GLP-1RA, glucagon-like peptide-1 receptor antagonist; sd, standard deviation; MAFLD, metabolic dysfunction-associated fatty liver disease; SGLT2i, sodium glucose cotransporter-2 inhibitor; SU, sulfonylurea; TZD, thiazolidinedione
- cell suppressed as number <11
†Others include Native American, Native Alaskan, Native Hawaiian
‡obtained only from patients with available lab values
§BMI and obesity information was not available in all patients
*eGFR calculated from MDRD race-free equation
#includes phentermine, topiramate, naltrexone, bupropion, benzphetamine, diethylpropion, phendimetrazine, orlistat
